# Virulence Role of the GlcNAc Side Chain of the Lancefield Cell Wall Carbohydrate Antigen in Non-M1-Serotype Group A *Streptococcus*

**DOI:** 10.1128/mBio.02294-17

**Published:** 2018-01-30

**Authors:** Anna Henningham, Mark R. Davies, Satoshi Uchiyama, Nina M. van Sorge, Sean Lund, Kelsey T. Chen, Mark J. Walker, Jason N. Cole, Victor Nizet

**Affiliations:** a Department of Pediatrics, University of California, San Diego, California, USA; b School of Chemistry and Molecular Biosciences and Australian Infectious Diseases Research Centre, University of Queensland, Brisbane, Queensland, Australia; c Department of Microbiology and Immunology, Peter Doherty Institute for Infection and Immunity, University of Melbourne, Melbourne, Victoria, Australia; d Medical Microbiology, University Medical Center Utrecht, Utrecht, The Netherlands; e Skaggs School of Pharmacy and Pharmaceutical Sciences, University of California, San Diego, California, USA; KUMC

**Keywords:** group A carbohydrate, group A *Streptococcus*, Lancefield antigen, *Streptococcus pyogenes*, virulence factor, innate immunity

## Abstract

Classification of streptococci is based upon expression of unique cell wall carbohydrate antigens. All serotypes of group A *Streptococcus* (GAS; *Streptococcus pyogenes*), a leading cause of infection-related mortality worldwide, express the group A carbohydrate (GAC). GAC, the classical Lancefield antigen, is comprised of a polyrhamnose backbone with *N*-acetylglucosamine (GlcNAc) side chains. The immunodominant GlcNAc epitope of GAC is the basis of all rapid diagnostic testing for GAS infection. We previously identified the 12-gene GAC biosynthesis gene cluster and determined that the glycosyltransferase GacI was required for addition of the GlcNAc side chain to the polyrhamnose core. Loss of the GAC GlcNAc epitope in serotype M1 GAS resulted in attenuated virulence in two animal infection models and increased GAS sensitivity to killing by whole human blood, serum, neutrophils, and antimicrobial peptides. Here, we report that the GAC biosynthesis gene cluster is ubiquitous among 520 GAS isolates from global sources, representing 105 GAS *emm* serotypes. Isogenic Δ*gacI* mutants were constructed in M2, M3, M4, M28, and M89 backgrounds and displayed an array of phenotypes in susceptibility to killing by whole human blood, baby rabbit serum, human platelet releasate, human neutrophils, and antimicrobial peptide LL-37. The contribution of the GlcNAc side chain to GAS survival *in vivo* also varied by strain, demonstrating that it is not a prerequisite for virulence in the murine infection model. Thus, the relative contribution of GAC to virulence in non-M1 serotypes appears to depend on the quorum of other virulence factors that each strain possesses.

## IMPORTANCE

The Lancefield group A carbohydrate (GAC) is the species-defining antigen for group A *Streptococcus* (GAS), comprising ~50% of the cell wall of this major human pathogen. We previously showed that the GlcNAc side chain of GAC contributes to the innate immune resistance and animal virulence phenotypes of the globally disseminated strain of serotype M1 GAS. Here, we use isogenic mutagenesis to examine the role of GAC GlcNAc in five additional medically relevant GAS serotypes. Overall, the GlcNAc side chain of GAC contributes to the innate immune resistance of GAS, but the relative contribution varies among individual strains. Moreover, the GAC GlcNAc side chain is not a universal prerequisite for GAS virulence in the animal model.

## INTRODUCTION

The Gram-positive human pathogen *Streptococcus pyogenes* is the single species comprising the group A *Streptococcus* (GAS). GAS infection can lead to mild or invasive disease, and each year GAS is responsible for ~700 million cases of superficial skin (impetigo) and throat (pharyngitis) infections and ~650,000 cases of severe invasive infections (such as bacteremia/sepsis, necrotizing fasciitis, and streptococcal toxic shock syndrome), of which ~25% are reported to be fatal (1, 2). GAS is ranked among the top 10 human pathogens causing infection-related deaths (1), placing a significant economic and health burden on public health systems worldwide. GAS exhibits high serotype diversity, which depends on a unique surface-expressed M protein that varies in its N-terminal hypervariable region, with over 200 serotypes reported (3). M protein is a multifunctional protein in which properties and virulence roles differ with serotype. M proteins can bind host fibrinogen and fibronectin (4) and plasminogen (5, 6) and interfere with complement deposition by binding the Fc domains of IgG and the complement-regulatory proteins C4BP and factor H (7, 8), thereby resisting opsonophagocytosis (9). Of the many disparate serotypes, M1 GAS is the most frequently isolated serotype from cases of invasive human disease occurring in high-income countries (10).

The serological classiﬁcation of streptococcal species depends upon the expression of cell wall-anchored carbohydrates in the bacterial cell wall (11). These cell wall carbohydrates play a structural role in streptococcal cell wall biogenesis (12, 13). All serotypes of GAS, irrespective of which M protein they produce, express the group A carbohydrate (GAC), which is comprised of a polyrhamnose core with an immunodominant *N*-acetylglucosamine (GlcNAc) side chain (13, 14). In the clinic, rapid diagnostic testing for GAS infection is based on the agglutination of antibody-coated latex beads that interact with the GAC GlcNAc epitope. The surface exposure of GAC on the cell wall, in addition to its ubiquitous expression, prompted the consideration of GAC as a GAS vaccine antigen. Indeed, administration of purified or synthetic native GAC conjugated to protein carriers provides protection in mice against infection with multiple GAS serotypes (15, 16). However, safety concerns regarding cross-reactivity of antibodies raised against the GAC GlcNAc side chain with GlcNAc epitopes present in host tissues (17–20) limit the use of native GAC in GAS vaccine preparations.

The GAS cell surface contains an intricate array of virulence factors and molecules, which contribute to adherence to and invasion of host cells and evasion of innate immune responses. One such molecule is the immunologically inert hyaluronan (HA) capsule, comprised of alternating glucuronic acid and GlcNAc residues. HA capsule obstructs antibody binding, complement deposition, and opsonophagocytosis (21, 22) and contributes to GAS colonization of pharyngeal cells (23, 24) and invasive infections (25, 26). Some serotypes, including M4 GAS, lack the *hasABC* operon that encodes the HA capsule but still remain virulent (27, 28). Other surface-associated virulence factors include adhesins such as pili (29), fibronectin-binding proteins (see the work of Walker et al. [2] for a comprehensive list), collagen-like proteins (30, 31), laminin-binding proteins (32, 33), and plasminogen-binding proteins (34, 35), all of which mediate adherence to host proteins and tissues. Other surface-associated virulence factors enable GAS to circumvent the host innate immune response by degrading chemokines (SpyCEP [36] and C5a peptidase [37]) and neutrophil extracellular traps (NETs) (Sda1 [38]), conferring antimicrobial peptide resistance (SpeB [39] and SIC [40]), impairing phagocytic uptake (IdeS/Mac-1 [41] and Mac-2 [42]), interfering with complement deposition (HA [21] and M protein [7, 43]), degrading antibodies (EndoS [44], Mac-1/2 [42, 45], and SpeB [46]), or binding antibodies nonspecifically (M protein [47], protein H [48], and SfbI [49]). Each of these mechanisms has been extensively reviewed elsewhere (2). It is assumed that every individual serotype/strain of GAS expresses a unique repertoire of such surface-associated virulence factors, which together allow infection in the host and promote innate immune resistance.

Recently, we identified the 12-gene GAC biosynthesis gene cluster and uncovered a novel role of the GAC GlcNAc side chain in the virulence of M1 GAS (50). Expression of the glycosyltransferase GacI, which is encoded by the *gacI* gene, was determined to be critical for the addition of the GlcNAc side chain to the polyrhamnose core of the GAC. Loss of the GAC GlcNAc epitope in serotype M1 GAS attenuated virulence in two animal infection models and increased GAS sensitivity to killing by blood, serum, platelet-derived antimicrobials, neutrophils, NETs, and cationic antimicrobial peptide LL-37. In this study, we set out to clarify the relative contribution of the GAC GlcNAc side chain to the pathogenicity and innate immune resistance of five non-M1 GAS serotypes.

## RESULTS

### The GAC biosynthesis gene cluster is ubiquitous among different GAS serotypes.

**FIG 1:**
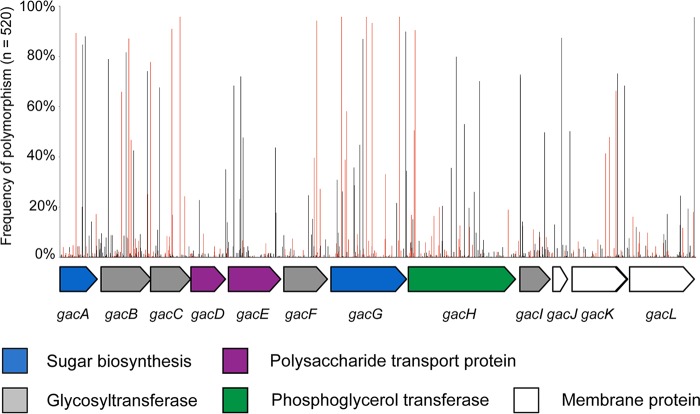
Genetic polymorphisms within the GAC biosynthesis gene cluster from 520 GAS genome sequences. Schematic representation of the 14,279-bp, 12-gene GAC gene cluster from the MGAS5005 type M1 (65, 67) with purported roles of gene product provided beneath. Shown above the schematic are the relative positions of 848 single nucleotide polymorphisms identified from BlastN analyses as a percentage of 520 GAS genome sequences screened. Black bars refer to synonymous polymorphisms (resulting in no amino acid change), while red bars refer to nonsynonymous polymorphisms (alteration of amino acid sequence) relative to the MGAS5005 reference sequence. Refer to Table S2 for specific polymorphism positions.

Allelic variation within the 12-gene *gac* gene cluster was examined by BlastN analysis against a database of 520 genome sequences, including 24 completely sequenced reference GAS genomes and additional draft genome sequences from Canada (51), Kenya (52), Lebanon (53), and Hong Kong (54, 55). The genome database was comprised of 105 known *emm* sequence types, 30 *emm* clusters (56), and 180 multilocus sequence types, reflecting a high representation of strain diversity as defined by standard GAS typing methodologies. All 520 genome sequences had over 99% homology to the MGAS5005 *gac* gene cluster, suggesting an overall high level of conservation within the gene cluster. A total of 848 single nucleotide polymorphic sites were identified within the 14,279-bp *gac* gene cluster across all 520 genomes with an average of 55 polymorphisms per genome across non-M1 genome sequences (range of 42 to 91 single nucleotide polymorphisms [SNPs] [see Table S1 in the supplemental material]). Eight hundred twenty-one SNPs (97%) were located within *gac* coding sequences, of which 295 (35%) were nonsynonymous, resulting in amino acid changes, and 426 were synonymous (50%, no amino acid change) (Fig. 1; Table S2). Three SNPs were predicted to result in premature stop codons within *gacC* (*n* = 1), *gacH* (*n* = 2), and *gacL* (*n* = 1) and thus are likely pseudogenes (Table S2). The average ratio of synonymous to nonsynonymous SNPs within the GAC operon was 0.24. In comparison, the average rate of synonymous to nonsynonymous SNPs within the hyaluronic acid capsule, the *hasABC* synthase operon, averaged 0.55 (Table S1).

10.1128/mBio.02294-17.2 TABLE S1  Metadata of 520 GAS genome sequences screened in this study. Download TABLE S1, XLSX file, 0.04 MB. Copyright © 2018 Henningham et al. 2018 Henningham et al. This content is distributed under the terms of the Creative Commons Attribution 4.0 International license.

10.1128/mBio.02294-17.3 TABLE S2  Location and property of 848 single nucleotide polymorphism sites within 520 GAS GAC operons. Download TABLE S2, XLSX file, 1.2 MB. Copyright © 2018 Henningham et al. 2018 Henningham et al. This content is distributed under the terms of the Creative Commons Attribution 4.0 International license.

### Non-M1-serotype Δ*gacI* mutants have lost the GlcNAc side chain from GAC.

**FIG 2:**
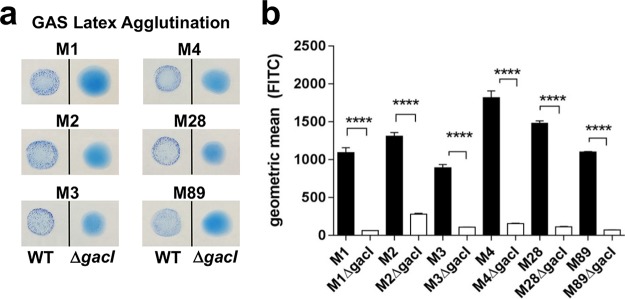
Δ*gacI* mutants do not express GlcNAc. (a) Latex agglutination reaction with GAC-speciﬁc beads. (b) GlcNAc-speciﬁc sWGA lectin staining of WT (black) and Δ*gacI* (white) strains. Two independent replicates containing triplicate samples were prepared, and representative data are presented (mean ± standard error of the mean [SEM]). Significant differences (*P* < 0.0001; denoted by ****) as determined by an unpaired Student *t* test are indicated.

Previously, the 12-gene GAC biosynthesis locus was identified and characterized in serotype M1 GAS, and an isogenic *gacI* mutant lacking the glycosyltransferase GacI was defective for GlcNAc side chain addition in the M1 genetic background (50). We set out to characterize the importance of the GAC GlcNAc side chain and the relative contribution to innate immune resistance in non-M1 serotypes of GAS. Following the generation of precise in-frame allelic replacement mutants eliminating the *gacI* gene in M2, M3, M4, M28, and M89 serotype GAS, each Δ*gacI* mutant lost reactivity in the diagnostic GAS latex agglutination test (Fig. 2a). When tested with the GlcNAc-speciﬁc lectin succinylated wheat germ agglutinin (sWGA), each Δ*gacI* mutant bound significantly less lectin than the respective wild-type (WT) strain (Fig. 2b). As was the case in the M1 genetic background, deletion of *gacI* in other GAS serotypes resulted in loss of the GAC GlcNAc side chain.

### Non-M1-serotype Δ*gacI* mutants are not impaired in growth, localization of the M protein, or production of the HA capsule.

Each of the isogenic Δ*gacI* mutants was compared to its respective WT parent strain to examine possible phenotypic and functional consequences arising following the loss of the GAC GlcNAc side chain. When tested in bacteriologic broth, the growth of each non-M1-serotype Δ*gacI* mutant did not significantly differ from the growth of the respective WT strain (Fig. S1a). Two important virulence factors in GAS are the surface-anchored M protein and the HA capsule. When assessing each of the non-M1-serotype Δ*gacI* mutants alongside its respective WT parent strain, there was no significant difference in the expression of M protein (Fig. S1b) or in the production of HA capsule (Fig. S1c). Consistent with previous studies (27, 28), the GAS M4 serotype did not express HA capsule. Serotype M28 was also lacking HA capsule. A recent publication shows that the *hasABC* genes are present in M28 GAS, but there is a frameshift mutation disrupting the coding sequence of *hasA*, the hyaluronan synthase gene, which is essential for hyaluronan synthesis, which is likely responsible for this finding (57). Thus, the non-M1-serotype Δ*gacI* mutants are not impaired in growth or expression of important virulence factors.

10.1128/mBio.02294-17.1 FIG S1  Δ*gacI* mutants do not have impaired expression of major GAS virulence factors. (A) Growth of WT (black) and Δ*gacI* (white) strains in Todd-Hewitt broth, measuring optical density at 600 nm. Two independent experiments containing duplicate samples were prepared, and data from the two experiments were pooled (mean ± SEM). (B and C) M protein surface expression (B) and hyaluronan capsule expression (C) in WT (black) and Δ*gacI* (white) strains. Pooled normalized data from two independent experiments containing triplicate samples are shown (mean ± SEM; unpaired Student’s *t* test; *P* < 0.05). Download FIG S1, TIF file, 0.8 MB. Copyright © 2018 Henningham et al. 2018 Henningham et al. This content is distributed under the terms of the Creative Commons Attribution 4.0 International license.

### The contribution of the GAC GlcNAc side chain to the survival of GAS grown in the presence of whole blood, serum, neutrophils, and antimicrobial peptides.

**FIG 3:**
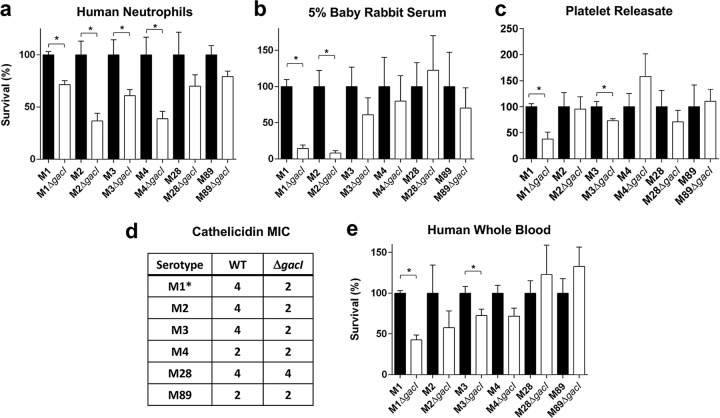
Contribution of GAC GlcNAc side chain to GAS innate immune resistance in non-M1-serotype GAS. (a) Survival of WT (black) and Δ*gacI* (white) strains in isolated human neutrophils at a multiplicity of infection of 0.1; surviving CFU were quantified after 30 min (*P* < 0.05; indicated by an asterisk). (b) Survival in 5% baby rabbit serum; survival was quantified after a 6-h incubation at 37°C (*P* < 0.01; indicated by an asterisk). (c) Survival in platelet releasate; surviving CFU were quantified after 2 h (*P* < 0.05; indicated by an asterisk). (d) Cathelicidin human antimicrobial peptide LL-37 micromolar MICs of the WT and Δ*gacI* strains are displayed (*t* = 24 h); *, M1 data are from the work of van Sorge et al. (50). (e) Survival in whole human blood isolated from a healthy donor at the 30-min time point (*P* < 0.05; indicated by an asterisk). The survival of each WT strain has been normalized to 100%. Pooled normalized data from two independent experiments containing triplicate samples are shown (mean ± SEM; unpaired Student’s *t* test).

In a previous study, the M1 GAS Δ*gacI* mutant displayed increased sensitivity to killing by human whole blood, neutrophils, and platelet-derived antimicrobials in serum and to the cathelicidin antimicrobial peptide LL-37 (50). Here, we assessed the role of the GAC GlcNAc side chain in promoting innate immune survival in non-M1-serotype GAS. Following the growth of each of the non-M1-serotype Δ*gacI* mutants and the respective WT parent strains in whole human blood, the M3 Δ*gacI* mutant was significantly more susceptible to killing (Fig. 3a). The M2, M4, M28, and M89 Δ*gacI* mutants did not significantly differ in their capacity to survive in human blood, compared to their respective WT parent strains (Fig. 3a). In contrast, the M1 Δ*gacI* mutant exhibited reduced blood survival compared to WT.

We next assessed the capacity of the non-M1-serotype Δ*gacI* mutants to survive in individual blood fractions and components such as purified neutrophils, serum, platelet-derived antimicrobials (or platelet releasate), and antimicrobial peptide LL-37. When incubated with freshly isolated human neutrophils, like the M1 Δ*gacI* mutant, the M2, M3, M4, and M89 Δ*gacI* mutants displayed attenuated growth compared to the respective WT parent strains (Fig. 3b). M28 was the only serotype in which the Δ*gacI* mutant did not exhibit significantly increased sensitivity to neutrophil-mediated killing in comparison to the WT. When incubated in serum (baby rabbit), compared to each of the WT parent strains, only the M2 Δ*gacI* mutant was significantly attenuated for growth, similarly to the M1 Δ*gacI* mutant (Fig. 3c). The survival of the M3, M4, M28, and M89 Δ*gacI* mutants in serum was not significantly different from that of the respective WT parent strains (Fig. 3c). When incubated with freshly isolated platelet-derived antimicrobials, only the M3 Δ*gacI* and M1 Δ*gacI* mutants were significantly attenuated for growth, compared to the WT parent strain (Fig. 3d). The survival of the M2, M4, M28, and M89 Δ*gacI* mutants in platelet-derived antimicrobials did not significantly differ from that of the respective WT parent strains (Fig. 3d). Finally, the MIC of human cathelicidin LL-37 was determined for each of the non-M1-serotype Δ*gacI* mutants. The M2 and M3 Δ*gacI* mutants had an LL-37 MIC that was half that of their respective WT strains (Fig. 3e), suggesting that they were more sensitive to killing by LL-37 than WT. The LL-37 MICs for M4, M28, and M89 Δ*gacI* mutants did not differ compared to the respective WT parent strains (Fig. 3e).

### The contribution of the GAC GlcNAc side chain to GAS virulence in a murine systemic infection model.

**FIG 4:**
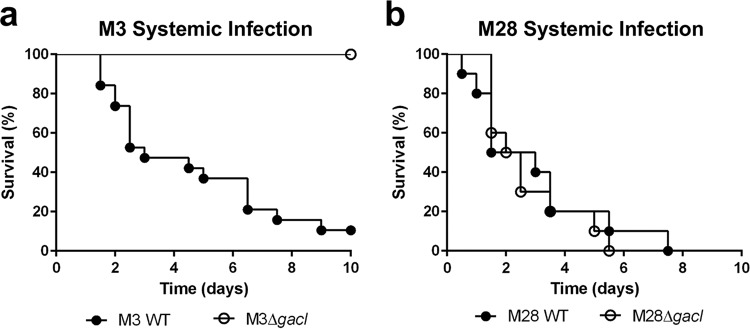
Contribution of GAC GlcNAc side chain to the virulence of non-M1-serotype GAS in a systemic mouse infection model. Survival curves for CD-1 mice following systemic (i.p.) infection with GAS WT (filled symbols) or Δ*gacI* mutant (open symbols) bacteria; survival was monitored for 10 days (log rank test). (a) Serotype M3 GAS, dose = 1 × 10^8^ to 2 × 10^8^ CFU (*P* < 0.0001). For M3 WT, *n* = 19 mice were used, and for M3 Δ*gacI*, *n* = 10 mice were used. (b) Serotype M28 GAS, dose = 4.5 × 10^8^ to 6 × 10^8^ CFU (not significantly different as *P* > 0.05). For both M28 WT and M28 Δ*gacI*, *n* = 20 mice were used.

After considering the results of the *in vitro* assays (Table S3), M3 and M28 were selected as two representative serotypes for further testing in a murine model of systemic infection. After observing the M3 Δ*gacI* mutant to be attenuated for survival in whole blood, platelet releasate, and isolated neutrophils, we hypothesized that the M3 Δ*gacI* mutant would likely be attenuated *in vivo*. In contrast, we hypothesized that the M28 Δ*gacI* mutant would likely not be attenuated *in vivo*, as it was not attenuated in any of the *in vitro* assays. Following infection of CD-1 mice via the intraperitoneal (i.p.) route with a lethal dose of GAS, the M3 Δ*gacI* mutant was observed to be attenuated for virulence compared to the WT (Fig. 4a) (*P* < 0.0001), whereas there was no difference in virulence when mice were infected with WT M28 or the M28 Δ*gacI* mutant (Fig. 4b) (*P* = 0.7038). These results indicate that the GAC GlcNAc side chain is not a universal prerequisite for virulence in the animal model and may parallel its contribution to resistance to innate immune factors in *ex vivo* assays (e.g., whole-blood killing).

10.1128/mBio.02294-17.4 TABLE S3  Summary of Δ*gacI* mutant phenotypes in innate immunity *in vitro* assays. Download TABLE S3, DOCX file, 0.01 MB. Copyright © 2018 Henningham et al. 2018 Henningham et al. This content is distributed under the terms of the Creative Commons Attribution 4.0 International license.

## DISCUSSION

GAC was first described over 60 years ago, and the GlcNAc side chain of GAC has been routinely utilized in the clinic as the key diagnostic epitope for GAS infection. The GAC has long been known to be an essential structural component of the GAS cell wall (13). However, it was only recently that the 12-gene GAC biosynthesis gene cluster was identified and that a fundamental role of the GAC GlcNAc side chain in virulence was described (50). In serotype M1 GAS, the loss of the GAC GlcNAc side chain resulted in decreased survival in human blood and systemic animal infection models (50). The relative contribution of the GAC GlcNAc side chain to the virulence and innate immune evasion of non-M1 GAS was unknown and has been characterized in this study. Overall, loss of the GlcNAc side chain increased susceptibility to diverse innate immune mechanisms in a strain-specific manner. In the M3 background, the GlcNAc side chain promoted survival in human whole blood and in the presence of platelet releasate, whereas the M2 Δ*gacI* mutant was attenuated in serum. Resistance to neutrophil killing was a more conserved trait associated with the GAC GlcNAc side chain observed to contribute to resistance in the M1, M2, M3, and M4 GAS strains. Possibly, increased killing by neutrophils of the M2 and M3 Δ*gacI* strains may be linked to increased susceptibility to cathelicidin LL-37. For GAS M28, no difference in survival between the Δ*gacI* mutant and WT was observed in any *in vitro* assays. Correspondingly, the M28 Δ*gacI* mutant was not attenuated in virulence compared to WT M28 in a systemic mouse infection model. While there was no significant difference in phenotype between the WT and the Δ*gacI* mutant in our mouse model, we cannot exclude the possible attenuation of the M28 Δ*gacI* mutant in other murine infection models or humans, the natural host of GAS. As our approved animal protocols follow NIH ethical guidelines to reduce overall mouse numbers used in experiments, we selected one representative strain with an attenuated Δ*gacI* mutant phenotype *in vitro* (M3) and one strain in which the Δ*gacI* phenotype was not attenuated *in vitro* (M28); we conclude that GAC does not contribute to virulence in all GAS strains equally.

The genes comprising the GAC biosynthesis gene cluster, *gacA* to *gacL*, are ubiquitous among a panel of 520 global GAS genome sequences representing over half of the known GAS *emm* sequence types. This highlights the fact that the entire 12-gene *gac* gene cluster forms part of the core GAS genome and is found as a single genomic gene cluster. A recent study of 328 GAS genome sequences from Kenya identified that each genome had an average of 1 polymorphism every 123 bp relative to the “core” MGAS5005 genome (1,629,062 bp) (52). In comparison, the GAC gene cluster from the same 328 Kenya genomes reported in this study had an average of 1 polymorphism per 260 bp, suggesting that sequence variation in the GAC gene cluster may be negatively selected. Indeed, the *gac* operon had a lower ratio of nonsynonymous to synonymous SNPs than did the HA capsule *hasABC* operon.

Many GAS surface proteins, antigens, and molecules exhibit differential carriage within and between *emm* sequence types, especially phage-associated determinants (56). For instance, M4 and M22 GAS completely lack the HA capsule, as they do not contain the *hasABC* operon (27, 28). Furthermore, among the remaining GAS serotypes that express HA capsule, expression levels can vary considerably, with mucoid isolates expressing copious quantities of capsule. GAS can undergo a genetic switch to a hypervirulent phenotype, in which the two-component regulator *covRS* undergoes spontaneous mutation, altering the expression of approximately 10 to 15% of the genome, including an upregulation of *hasA* leading to hyperencapsulation (58). The carriage of surface-expressed fibronectin-binding proteins also varies widely between GAS serotypes. PrtF1/SfbI and PrtF2/PFBP/FbaB are expressed only in FCT [fibronectin-collagen-T-antigen]-specific GAS strains (59), SOF/SfbII (60) and SfbX (61) are present in only 55% of strains, and to date, FbaA has been reported in only 18 serotypes (62). Thus, the context and surface accessibility of GAC will vary across GAS strains, which may influence its interaction with host cells and soluble factors and consequently its potential to influence immune resistance phenotypes. Additionally, the presence of sufficient additional GAS immune resistance factors may provide functional redundancy to specific functions of the GAC GlcNAc side chain among GAS serotypes/strains. Thus, one can envisage GAC being present among a unique, intricate network of surface proteins and molecules displayed on the surface of each GAS strain.

While this study focused on strains that lack the glycosyltransferase *gacI*, a recent paper has characterized *gacA*, the first gene in the GAC gene cluster (63). GacA was determined to be an essential enzyme, functioning in a novel monomeric manner to catalyze the ﬁnal step of the four-step dTDP–l-rhamnose biosynthesis pathway during the production of the GAC polyrhamnose core. van der Beek and colleagues (63) suggest targeting the nonmammalian l-rhamnose biosynthesis mediated by GacA as a potential strategy for the development of novel antimicrobial compounds against GAS. The characterization of the remaining genes in the GAC biosynthesis gene cluster may reveal additional enzymes that could function as valid antimicrobial drug targets. In contrast to many existing antibiotics which function to kill bacteria, the direct targeting of essential enzymes, such as those in the GAC gene cluster, may “disarm” the pathogen, render it harmless, and allow the body’s natural defenses to eliminate the pathogen and clear the infection (64).

Overall, the GlcNAc side chain of GAC does contribute to the innate immune resistance of GAS, but the relative contribution varies among the individual GAS strains. It is possible that differential phenotypes may be related to differential virulence gene carriage within the different isolates studied. Recent unbiased transposon-sequencing (Tn-seq) screens for GAS M1T1 genes essential for *in vitro* viability (64) or *in vivo* fitness during skin infection (65) independently corroborated the essentiality of the polyrhamnose backbone and the virulence function of the GlcNAc side chain (*gacI*) established in our original characterization of the operon (50). Furthermore, the molecular mechanism by which GAS attaches GlcNac to the polyrhamnose via two distinct undecaprenol-linked GlcNAc-lipid intermediates has now been deduced, indicating that the side chain protects GAS from amidase-induced lysis (66). However, the present study across different serotype strains concludes that the GAC GlcNAc side chain is not a universal GAS virulence factor. The relative contribution of the GAC GlcNAc side chain to virulence in non-M1 serotypes appears to be dependent on the quorum of other virulence factors that each strain possesses. It is likely that the abundance of virulence factors expressed or secreted from the surface of GAS can compensate for the loss of the GlcNAc side chain in some strain backgrounds.

## MATERIALS AND METHODS

### Genomic screening of the GAC gene cluster.

The 14,279-bp *gac* gene cluster (*gacA* to *gacL*) from the resequenced MGAS5005 type M1 genome sequence (67) was used as the reference sequence for *gac* diversity analyses. A panel of GAS genome sequences, including 24 published complete genome sequences and raw sequence data from the European Nucleotide Archive derived for GAS genetic diversity studies from Kenya (52), Canada (51), Hong Kong (54, 55), and Lebanon (53), was compiled to examine *gac* gene cluster diversity across *emm* sequence types and also geographic variation. Draft genome assemblies were generated using Velvet or an iterative assembler as used previously for the Kenyan (52) and Hong Kong (54, 55) genome sequences. GAS *emm* sequence type, multilocus sequence type, and *emm* cluster type (68) were determined by BLAST analyses of draft genome sequences and cross-referenced to published studies where applicable. Only genome assemblies less than 2.2 Mb and where the complete GAC gene cluster was unambiguously assembled into a single contig were used for allelic variation studies (a total of 520). Identification of the GAC gene cluster was determined by BlastN analyses of complete and draft GAS genome sequences at an E value cutoff of 1E−05 over 90% of the length of the *gac* gene cluster. Whole *gac* gene clusters were aligned using MUSCLE (69), and polymorphisms were identified from the resulting alignment.

### Bacterial strains and growth conditions.

GAS strain 5448, a representative of the globally disseminated serotype M1 clone, was isolated from a patient with toxic shock syndrome and necrotizing fasciitis (70). The isogenic 5448Δ*gacI* (50) and 5448Δ*emm*1 (71) mutants were described previously. Clinical GAS strains 3752-05 (*emm*2), 4041-05 (*emm*3), 4039-05 (*emm*28), and 4264-05 (*emm*89) were kindly provided by B. W. Beall (CDC, Atlanta, GA). GAS strain SP442 (*emm*4) was isolated from a child with suspected hand, foot, and mouth disease (27). GAS was routinely propagated at 37°C on Todd-Hewitt agar (THA) or in static liquid cultures of Todd-Hewitt broth (THB; Hardy Diagnostics). When necessary, growth medium was supplemented with erythromycin (Em) at 5 μg/ml or chloramphenicol (Cm) at 2 μg/ml. Unless indicated otherwise, logarithmic-growth-phase cultures with an optical density at 600 nm (OD_600_) of 0.4 were used for all experiments.

### Precise in-frame allelic exchange mutagenesis of *gacI*.

To construct GAS Δ*gacI* allelic exchange mutants in non-M1 serotypes of GAS, pHY304-*gacI*-KO, the *gacI* knockout plasmid (50), was transformed into wild-type (WT) M2, M3, M4, M28, or M89 GAS isolates by electroporation (72), and the transformants were selected by growth on THA-Em (5 μg/ml) for 2 days at 30°C. Single recombination events were selected for by shifting to the nonpermissive temperature (37°C) while maintaining Em selection. Selective pressure was relaxed by serial passage at 30°C without antibiotics, and double-crossover events were identified by screening for a Cm-resistant and Em-sensitive phenotype. The precise, in-frame allelic exchange of *gacI* with the *cat* gene in the non-M1 serotypes of GAS was verified by PCR using *gacI*- and *cat*-specific primers and the GAS latex agglutination assay described below. Non-M1 Δ*gacI* mutants were not complemented in this study as this has already been performed in the M1 serotype and shown to result in a complete restoration of all tested phenotypes (50).

### Latex agglutination assay.

Latex agglutination tests for GAS (Remel PathoDx) were performed according to the manufacturer’s instructions on overnight cultures.

### Growth curve analysis.

Overnight cultures of GAS were inoculated in fresh THB to an OD_600_ of 0.1. Two replicate tubes were incubated at 37°C under static conditions, with hourly measurements to monitor growth kinetics. Two independent experiments were performed, and the resultant growth curves for both experiments are presented.

### Lectin staining.

Overnight cultures were centrifuged and resuspended in HEPES++ buffer (20 mM HEPES, 140 mM NaCl, 5 mM CaCl_2_, 2.5 mM MgCl [pH 7.4]) plus 0.1% bovine serum albumin (BSA) (HEPES++ 0.1% BSA) to an OD_600_ of 0.4. The bacterial suspension (100 μl) was pelleted and stained with fluorescein isothiocyanate (FITC)-labeled succinylated wheat germ agglutinin (sWGA; Vector Laboratories) at a 1:2,500 dilution to assess GlcNAc expression as described previously (50). Staining was analyzed by ﬂow cytometry. Representative data are presented from 2 independent experiments.

### M protein expression.

Surface-localized M protein was quantiﬁed on mid-log bacterial cultures (OD_600_ of 0.4) using polyclonal mouse anti-M protein serum or naive mouse serum at a 1:1,000 dilution and Alexa 488-conjugated goat anti-mouse IgG secondary antibody at a 1:500 dilution (Life Technologies) as previously described (50). Staining was analyzed by ﬂow cytometry. Data were pooled and normalized from 2 independent experiments, each performed in duplicate. The 5448Δ*emm*1 mutant was included as a negative-control strain for antibody binding.

### Hyaluronan capsule quantification.

Hyaluronan capsule was extracted from GAS using chloroform as previously described (27) and quantified using the HA quantitative test kit (Corgenix), as previously described (58). Data were pooled and normalized from 2 independent experiments, each performed in triplicate.

### Whole-blood survival assays.

Whole-blood assays were performed as previously described (27). After 30 min, 25-μl aliquots were 10-fold serially diluted in phosphate-buffered saline (PBS), plated onto THA, and incubated at 37°C overnight for enumeration of surviving CFU. Percent survival was calculated by dividing the CFU at 30 min by the CFU at time zero, multiplied by 100%. Data were pooled and normalized from 2 independent experiments, each performed in triplicate.

### Serum survival assays.

Serum assays were performed as previously described (73) using baby rabbit serum (AbD Serotec). After 6 h, 25-μl aliquots were 10-fold serially diluted in PBS, plated onto THA, and incubated at 37°C overnight for enumeration of surviving CFU. Percent survival was calculated by dividing the CFU at 6 h by the CFU at time zero, multiplied by 100%. Data were pooled and normalized from 3 independent experiments, each performed in triplicate.

### Platelet releasate survival assay.

Platelets (2.4 × 10^8^ per ml) were stimulated with 3 U of bovine thrombin per ml for 25 min at 37°C as previously described (74). Following centrifugation (2,000 × *g*) for 10 min at 25°C, the releasate was recovered in the supernatant. Nonstimulated platelet releasates (without thrombin) were prepared in parallel, and 3 U of bovine thrombin per ml was added following the stimulation period, prior to the incubation with bacteria. Log-phase bacteria were resuspended in RPMI (without phenol red) to an OD_600_ of 0.4 and diluted 10-fold. A 10-μl volume of diluted bacteria (1 × 10^5^ CFU) was added to 90 μl of platelet releasate (either stimulated or nonstimulated) and incubated for 2 h at 37°C. Following the incubation, 25-μl aliquots were 10-fold serially diluted in PBS, plated onto THA, and then incubated overnight at 37°C for enumeration of CFU. Percent survival of the bacteria in the stimulated releasate was calculated in comparison to control wells grown with nonstimulated releasate. Data were pooled and normalized from 3 independent experiments performed in triplicate.

### Neutrophil killing assays.

Neutrophil killing assays were performed as previously described with a multiplicity of infection of 0.1 (27). Following incubation for 30 min, 25-μl aliquots were 10-fold serially diluted in molecular-grade water, plated onto THA, and incubated overnight at 37°C for enumeration of CFU. Percent survival of the bacteria was calculated in comparison to bacterial control wells grown under the same conditions in the absence of neutrophils. Data were pooled and normalized from 2 independent experiments performed in triplicate.

### LL-37 susceptibility.

LL-37 MICs were determined by incubating duplicate stationary-phase cultures in Dulbecco's modified Eagle medium (DMEM)-10% THB with various concentrations of LL-37 (4, 2, 1, 0.5, or 0 µm LL-37) for 24 h at 37°C. Growth was recorded by measuring OD_600_ every 30 min for 24 h using the Bioscreen C MBR system. The MIC was defined as the concentration of LL-37 which did not allow growth of the strain over the 24-h period.

### Systemic infection model.

Groups of 8-week-old female CD-1 mice (Charles River Laboratories, Inc.) were inoculated intraperitoneally (i.p.) with ~10^8^ CFU of GAS (WT or Δ*gacI* mutant) in 200 μl of PBS containing 5% porcine gastric mucin (Sigma). Survival was monitored twice daily for 10 days. The number of mice used for each strain was as follows: M3 WT, 19 mice; M3 Δ*gacI* mutant, 10 mice; M28 WT and M28 Δ*gacI* mutant, both 20 mice.

### Statistical analyses.

For lectin staining, M protein surface expression, hyaluronan capsular expression levels, whole-blood survival, serum survival, platelet releasate survival, and neutrophil survival, each WT and Δ*gacI* mutant pair were compared by unpaired Student’s *t* test. Differences were considered significantly different at a *P* value of <0.05. All statistical analyses were performed using GraphPad Prism version 5.0b (GraphPad Software, Inc.).

### Ethics approvals.

Permission to collect human blood under informed consent was approved by the UCSD Human Research Protection Program. Procedures used for all animal experiments were approved by the UCSD Institutional Animal Care and Use Committee.
