# Supplementary figures and images for: Virulence Role of the GlcNAc Side Chain of the Lancefield Cell Wall Carbohydrate Antigen in Non-M1-Serotype Group A Streptococcus
Source: mBio. 2018 Jan 30;9(1):e02294-17. doi: 10.1128/mBio.02294-17 (PMC5790915; doi:10.1128/mBio.02294-17)

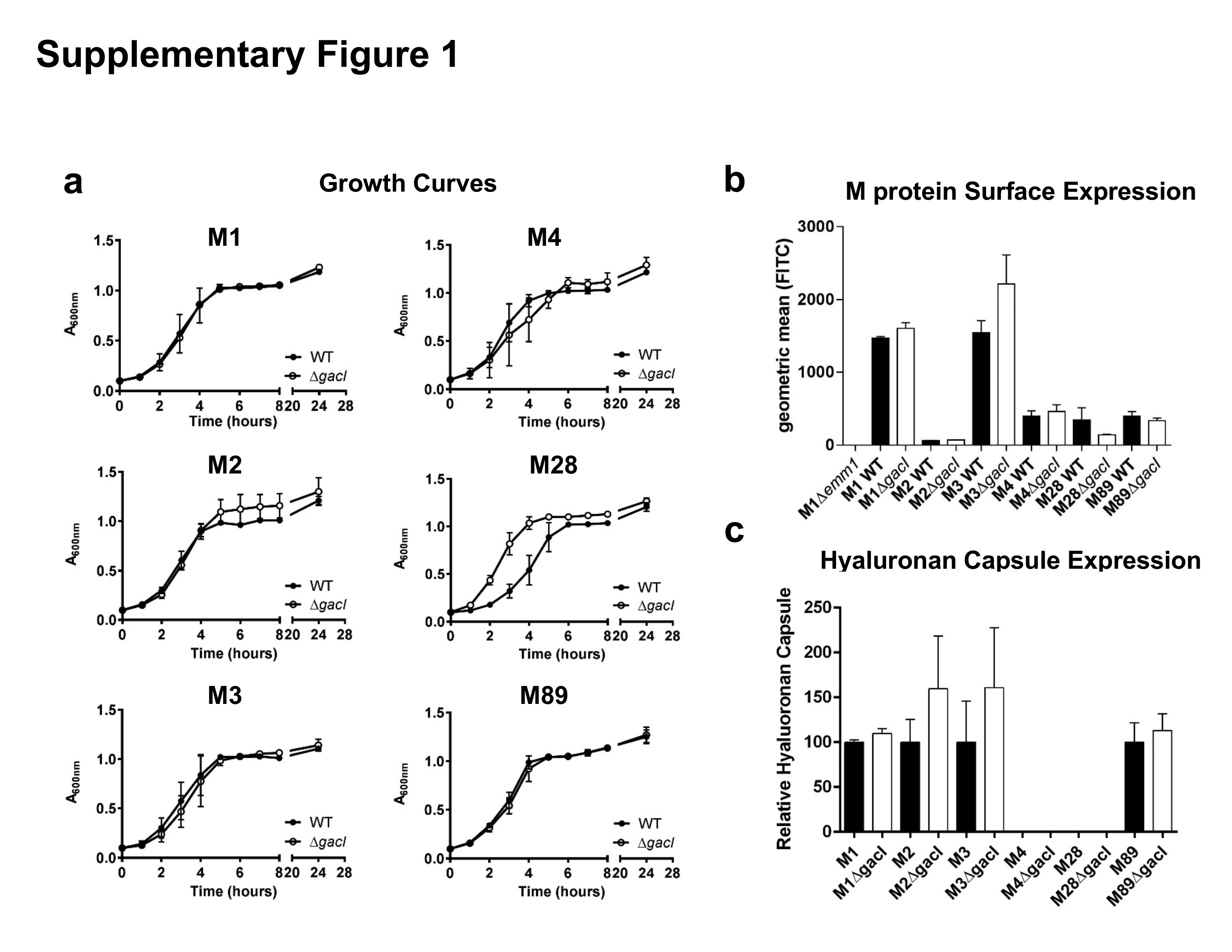

Supplement: FIG S1 [file mbo001183702sf1.tif]
