# Supplementary material for: Virulence Role of the GlcNAc Side Chain of the Lancefield Cell Wall Carbohydrate Antigen in Non-M1-Serotype Group A Streptococcus
Source: mBio. 2018 Jan 30;9(1):e02294-17. doi: 10.1128/mBio.02294-17 (PMC5790915; doi:10.1128/mBio.02294-17)
Supplement: TABLE S3 [file mbo001183702st3.docx]

**Supplementary Table 3:** Summary of **Δ***gacI* mutant phenotypes in innate immunity *in vitro* assays.

|  | **M1Δ*gacI*** | **M2Δ*gacI*** | **M3Δ*gacI*** | **M4Δ*gacI*** | **M28Δ*gacI*** | **M89Δ*gacI*** |
| --- | --- | --- | --- | --- | --- | --- |
| **Blood** | + | - | + | - | - | - |
| **Serum** | + | + | - | - | - | - |
| **Platelet Releasate** | + | - | + | - | - | - |
| **Neutrophils** | + | + | + | + | - | + |
| **LL-37** | + | + | + | - | - | - |

+Indicates significant difference compared to parent WT strain; -, indicates no significant difference.
